# The effectiveness of acupuncture research across components of the trauma spectrum response (tsr): a systematic review of reviews

**DOI:** 10.1186/2046-4053-1-46

**Published:** 2012-10-15

**Authors:** Courtney Lee, Cindy Crawford, Dawn Wallerstedt, Alexandra York, Alaine Duncan, Jennifer Smith, Meredith Sprengel, Richard Welton, Wayne Jonas

**Affiliations:** 1 Samueli Institute, 1737 King Street, Suite 600, Alexandria, VA, USA; 2 Integrative Healing, 8505 Fenton Street, Suite 202, Silver Spring, Maryland, USA

**Keywords:** Acupuncture, Trauma Spectrum Response, Systematic review of reviews, Rapid evidence assessment of the literature (REAL©)

## Abstract

**Background:**

Co-morbid symptoms (for example, chronic pain, depression, anxiety, and fatigue) are particularly common in military fighters returning from the current conflicts, who have experienced physical and/or psychological trauma. These overlapping conditions cut across the boundaries of mind, brain and body, resulting in a common symptomatic and functional spectrum of physical, cognitive, psychological and behavioral effects referred to as the ‘Trauma Spectrum Response’ (TSR). While acupuncture has been shown to treat some of these components effectively, the current literature is often difficult to interpret, inconsistent or of variable quality. Thus, to gauge comprehensively the effectiveness of acupuncture across TSR components, a systematic review of reviews was conducted using the Samueli Institute’s Rapid Evidence Assessment of the Literature (REAL©) methodology.

**Methods:**

PubMed/MEDLINE, the Cochrane Database of Systematic Reviews, EMBASE, CINAHL, and PsycInfo were searched from inception to September 2011 for systematic reviews/meta-analyses. Quality assessment was rigorously performed using the Scottish Intercollegiate Guidelines Network (SIGN 50) checklist and the Grading of Recommendations, Assessment, Development and Evaluation (GRADE) methodology. Adherence to the Standards for Reporting Interventions in Clinical Trials in Acupuncture (STRICTA) criteria was also assessed.

**Results:**

Of the 1,480 citations identified by our searches, 52 systematic reviews/meta-analyses, all high quality except for one, met inclusion criteria for each TSR component except post-traumatic stress disorder (PTSD) and sexual function. The majority of reviews addressed most STRICTA components, but did not describe safety.

**Conclusions:**

Based on the results of our review, acupuncture appears to be effective for treating headaches and, although more research is needed, seems to be a promising treatment option for anxiety, sleep disturbances, depression and chronic pain. It does not, however, demonstrate any substantial treatment benefit for substance abuse. Because there were no reviews on PTSD or sexual function that met our pre-defined inclusion criteria, we cannot comment on acupuncture’s effectiveness in treating these conditions. More quality data are also needed to determine whether acupuncture is appropriate for treating fatigue or cognitive difficulties. Further, while acupuncture has been shown to be generally safe, safety was not described in the majority of studies, making it difficult to provide any strong recommendations. Future research should address safety reporting in detail in order to increase our confidence in acupuncture’s efficacy across the identified TSR components.

## Background

There is a complex interaction between pain, psychological distress and physical function [1-4]. Individuals with chronic pain often report depression [5], anxiety [6], sleep disturbance [7-9], fatigue [10], and changes in physical and cognitive functioning [11-14], mood [15], personality [16] and social relationships [17]. Research supports a positive association between a history of physical and/or psychological trauma and chronic pain [18-22] and depression [23]. Individuals with post-traumatic stress disorder (PTSD) have high rates of co-occurring conditions including suicide [24,25], substance abuse [26], anxiety [27], headache [28] and chronic pain [29,30], greater emotional distress [31], more interference with activities of daily living [32] and more pronounced disability than in pain patients without a history of trauma or PTSD [33].

**Figure 1:**
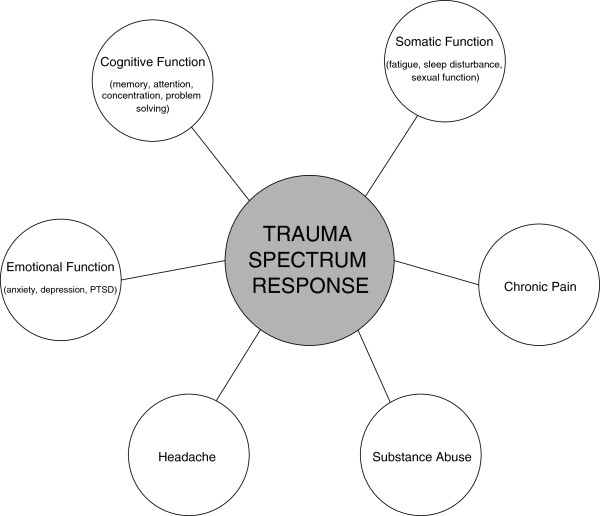
Trauma spectrum response.

These overlapping conditions, potentially triggered by combined mind-body/brain injuries have been termed the Trauma Spectrum Response (TSR) [29] and often include: (1) psychological and emotional distress (that is, depression, anxiety, PTSD); (2) cognitive impairment (for example, memory, attention); (3) chronic and, often refractory pain of organic and psychosomatic origins; (4) headache; (5) substance abuse; and (6) somatic dysfunction (that is, sexual function, fatigue, sleep disturbances; (Figure 1). Because the majority of individuals, after a psychological or physical injury, experience disturbances in one or more of these areas at varying degrees, the TSR, much like Seyle’s general stress response [34], is useful for understanding the whole person response to stress and injury.

Identifying appropriate self-management and treatment interventions for the TSR is critically important given the number of service members across the world returning from the Operation Enduring Freedom (OEF) and Operation Iraqi Freedom (OIF) conflicts with complex multi-symptom illness (CMI) [35] and functional disabilities, including traumatic brain injury (TBI), operational and/or combat stress, PTSD, and chronic pain.

The complex symptomatology of TSR is currently treated with a biomedical approach that usually provides isolated, specialized medical care for each symptom or diagnosis. Resulting medical procedures and poly-pharmacy treatment plans, however, may not fully address the TSR’s complexity and are frequently complicated by overlapping side-effects and difficulty in coordinating care [29]. Because such co-occurring conditions have been identified as bio-psychosocial determinants of persistent and chronic pain [36,37] in both trauma [38] and non-trauma populations [39], chronic pain may be better treated with a biopsychosocial approach that considers the complex interplay of physiological, psychosocial, environmental, cognitive, behavioral, and affective factors that influence an individual’s pain experience [40-42]. This whole-person approach can assess the ‘full spectrum of trauma-related morbidities (rather than individual components),’ in order to enhance the patient’s inherent healing mechanisms and capacities [29,43].

Acupuncture may be a promising whole-person treatment option for TSR as dynamic, ‘multi-mechanism’ responses are elicited by the insertion of thin needles at specific body points which influence several interacting pathways in trauma response and recovery [44]. Because acupuncture has been found to be effective in treating several individual conditions that form TSR (that is, insomnia [45-47], depression [48,49], chronic pain [50-52], headaches [53,54]) and other multiple co-morbidities [55]), it could be potentially advantageous for treating the multi-symptom complex of TSR. To date, however, no systematic reviews have examined acupuncture efficacy across the entire TSR. Although reviews on individual components of TSR exist, their methods vary, and their conclusions are often difficult to interpret, inconsistent or contradictory as they do not apply consistent methods. As such, we conducted a ‘systematic review of reviews’ using the Samueli Institute’s Rapid Evidence Assessment of the Literature (REAL©) methodology in order to achieve the following aims, developed in accordance with the PICO (that is, population, intervention, control, outcome) framework: (1) survey the available systematic reviews and meta-analyses to assess acupuncture efficacy comprehensively across the individual TSR components in all populations; (2) identify and summarize the quantity and quality of the published reviews; (3) describe the characteristics and safety issues of, and whether they adhere to, the Standards for Reporting Interventions in Clinical Trials of Acupuncture (STRICTA) criteria; and (5) identify gaps in research areas to guide a future research agenda.

## Methods

### Data sources and search strategy

The following databases were searched from their inception to September 2011: PubMed/MEDLINE, the Cochrane Database of Systematic Reviews, EMBASE, CINAHL, and PsycInfo to identify systematic reviews and meta-analyses on acupuncture as the intervention and each TSR component as an evaluated outcome. The authors explored MeSH (Medical Subject Headings) within MEDLINE to strategize the most powerful search and also consulted with three subject matter experts (SMEs; AY, AD, RW) to ensure that the correct key terms were being targeted for acupuncture and the various TSR components (see Table 1 for the full search strategy designed for the PubMed database). Variations of the search strategy for the remaining databases are available upon request from the primary author. Following traditional REAL© methodology which includes only peer-reviewed randomized controlled trials, systematic reviews or meta-analyses published in the English language, all searches were conducted with these specific limits, in addition to studies that involved human subjects. Where this was not a limit option in certain databases, citations were screened for these criteria.

**Table 1:** PubMed search strategy and keyword definitions

**Component**	**Search string**^**a**^	**Keywords**/**definitions for each component used in screening and review phases**
Chronic pain	Acupuncture AND (“pain”[Mesh] OR “pain” OR “chronic pain” OR “back pain”[Mesh] OR “facial neuralgia”[Mesh] OR “prostatitis”[Mesh] OR “fibromyalgia”[Mesh] OR “back pain*” OR “facial neuralgia*” OR “prostatitis” OR “fibromyalgia”)	defined as any condition included in the American Chronic Pain Association’s list of chronic conditions, or any type of pain lasting longer than three months
Substance abuse	Acupuncture AND (“substance-related disorders”[Mesh] OR “substance-related disorder” OR “drug dependence” OR “substance abuse”)	any drug or chemical abuse, dependence or addiction
Sleep disturbance	Acupuncture AND (“Sleep Disorders"[Mesh] OR “sleep disorder*”)	insomnia, narcolepsy, hyperarousal, sleep apnea
Depression	Acupuncture AND (“Depression”[Mesh] OR “Depressive Disorder”[Mesh] OR depression* OR “depressive disorder*”)	substance-induced mood disorder and major, chronic, bipolar, seasonal, psychotic, postpartum, double, secondary, chronic treatment-resistant, and masked depressions
Headache	Acupuncture AND (“Headache”[Mesh] OR “Headache Disorders”[Mesh] OR “headache*” OR “headache disorder*” OR “post-traumatic headache*”[Mesh] OR “post-concussive headache*” OR “post-concussive syndrome*” OR “TBI headache*”)	headache of any etiology and duration
Anxiety	Acupuncture AND (“Anxiety Disorders”[Mesh] OR “dental anxiety”[Mesh] OR “catastrophization”[Mesh] OR “anxiety disorder*” OR “anxiety”)	dissociative anxiety, generalized anxiety disorder, panic disorder, phobic disorder, obsessive compulsive disorder
Cognitive function	Acupuncture AND (“memory”[Mesh] OR “cognition”[Mesh] OR “memor*” OR “cognition*” OR “problem solving” OR “attention” OR “concentration”)	attention, concentration, memory, perception, and problem solving difficulties as well dementia, autism, attention deficit disorder, stroke
Fatigue	Acupuncture AND (“fatigue”[Mesh])	any type of fatigue
PTSD	Acupuncture AND (“Stress Disorders, Traumatic”[Mesh] or “post-traumatic stress disorder” OR “ptsd”)	any type of traumatic stress disorder
Sexual function	Acupuncture AND (“sexual function*” OR “sexual dysfunction*”)	any type of sexual function disorder

^a^keywords were searched using Mesh terms and automatic term mapping.

### Study selection

Articles were included if they met the following criteria: (1) systematic review or meta-analysis study design, with individual studies of any study design, presented in the English language and involving human subjects; (2) all populations being treated with acupuncture for at least one of the TSR components, as described in Table 1; (3) acupuncture involving needling of the skin at either recognized acupuncture or Ah Shi points; and (4) studies that used at least one outcome to measure a TSR component as described above.

Because broad search terms were used to be as comprehensive as possible for each TSR component, our searches would likely yield articles on a variety of conditions (for example, ‘sleep disturbance’ may yield articles on insomnia, narcolepsy, sleep apnea). To guide the team in screening articles for inclusion, our medical SME (RW), a retired Navy Captain and physician who has worked clinically with wounded service members, developed specific keywords and definitions; conditions considered to be sequelae of physical and/or psychological trauma were included in this review (see Table 1).

Acupuncturists often include additional interventions in their treatments, and recommend of self-management techniques (that is, skills that individuals can perform independently without requiring reliance on a trainer or therapist). These elements cannot be controlled and are often considered to be part of acupuncture treatment rather than adjunctive to acupuncture treatment. As such, we chose to include the following criterion, which was created to ensure that treatment effects were attributable only to acupuncture rather than to other interventions: acupuncture administered (a) alone; (b) with a co-intervention, defined as an intervention that was provided additionally to both treatment and control groups; or (c) with one of the following interventions, often considered to be part of acupuncture treatment: (i) techniques commonly used with acupuncture (for example, tui na, acupressure, cupping, moxibustion, herbs, tai qi, qi gung, gua sha, bleeding, Ah Shi points), and/or (ii) self-management techniques (for example, stretching exercises, meditation, tai chi, breathing exercises, suggested dietary/lifestyle changes).

Articles were excluded if they met at least one of the following criteria: (1) any study design other than a meta-analysis or systematic review; (2) the focus was on an intervention other than acupuncture; (3) acupuncture intervention did not involve needling of the skin at either recognized acupuncture or Ah Shi points (that is, TENS, laser acupuncture, dry needling or trigger point injections at non-acupuncture sites); (4) the acupuncture treatment plan included additional interventions/modalities that were not acupuncture-related or self-management techniques; (5) articles did not include at least three studies on a TSR-related outcome; and (6) quality assessment was not performed across the individual studies screened.

### Data extraction and quality assessment

We conducted this review using a web-based, secure, systematic review management program known as Mobius Analytics SRS (Copyright 2003–2009 Mobius Analytics Inc., Ottawa, ON, Canada), which automates article progression and management, eliminates data transcription and reduces post-review data collation and errors.

Two investigators (CL, JS) independently screened titles and abstracts for relevance based on the inclusion criteria pre-defined above. Any disagreements about inclusion were resolved either through discussion and consensus or by one of the SMEs. After this first, liberal screen, all articles marked for inclusion underwent a second level of screening due to the heterogeneity and abundance of reviews marked thus far for inclusion. The team felt it was important to ensure the final set of studies would be homogenous enough to be able to make definitive conclusions. As such, during this second screening phase, all articles with titles mentioning other interventions or complementary alternative therapies in general (that is, articles investigating the effect of various complementary and alternative medicine (CAM) therapies) were then excluded. Doing so would ensure that the focus was specific to the acupuncture intervention as defined by our inclusion criteria. All articles marked for inclusion after the second screen were retrieved and underwent a third level of screening during which one investigator screened the full text articles based on the inclusion criteria above for cross-checking.

Methodological quality was independently assessed by three reviewers (CL, JS, MS) for the individual studies included using the Scottish Intercollegiate Guidelines Network (SIGN 50) checklist for systematic reviews, a validated and reliable assessment approach widely used in the literature [56]. The following descriptive data were extracted: population, type of acupuncture, control intervention, all TSR-related outcome measures assessed, number of studies included, results of the individual reviews as reported by the authors, and whether the reviews reported on adverse events. Controls were grouped into the following categories: pharmacologic, no treatment (for example, standard care, wait list), placebo/sham acupuncture, CAM, behavioral/psychosocial (for example, rehabilitation, psychological therapy), acupuncture, physiological (for example, physical therapy, oxygen training), and other.

A rulebook that detailed how to extract this information and how to score the SIGN criteria objectively was developed by Samueli Institute researchers and was used to train the reviewers to ensure consistency in data extraction and reduce the number of errors and post-extraction data cleaning. Three reviewers, fully trained in this methodology, reviewed the articles in pairs until a sufficient kappa (>90%) was achieved, at which point they continued reviewing the remaining articles independently. All disagreements were resolved either through discussion and consensus, or by the SMEs.

Information regarding the criteria for the revised STRICTA guideline’s six-item checklist was also extracted. STRICTA, first published in 2001 and revised in 2010, is a formal extension of CONSORT, expanding the general content of item five (around the intervention description specific to acupuncture) of the CONSORT statement [57]. It was designed to improve the completeness of reporting interventions in controlled acupuncture trials so that such trials can provide a clear and accurate account of both acupuncture and control/comparison protocols. Because complete and accurate trial reports can facilitate dissemination, interpretation, translation and replicability, we noted whether or not authors included the information detailed in the STRICTA guidelines [58] in the included systematic reviews and meta-analyses. This would help us to determine where the gaps lay specific to acupuncture research.

### Data synthesis

Once the quality assessment of individual reviews was completed, two SMEs (AY, AD) examined the collected data and results of the individual reviews included to develop an ‘interpretation of the results’ by noting whether the individual reviews reported positive (favoring acupuncture), inconclusive (favoring neither acupuncture nor the comparison group), or negative (favoring the comparison group) results and whether the quality of the studies included in each review were of poor or strong methodology.

A meta-analysis was not conducted due to the heterogeneity across studies and lack of effect size reporting in the individual studies. Instead, the SMEs then performed a quality assessment of the overall literature pool for each component of TSR identified using a modified version of the Grading of Recommendations, Assessment, Development and Evaluation (GRADE) [59], an internationally accepted approach to grading the quality of evidence and strength of recommendations across studies. This GRADE was modified by the team to fit the parameters for evaluating systematic review results, and SMEs were trained in this methodology using a rulebook developed, tested and agreed upon by the entire team (available by contacting the primary author). Both SMEs examined the outcomes of the individual systematic reviews for each TSR component in order to: (1) examine the confidence in the results (using the same methodological criteria for GRADE but adapting the scoring to be: acupuncture shown to be effective; acupuncture promising, but no conclusions yet; unable to interpret/contradictory results; acupuncture shown to not be effective); (2) assign a safety grade to the literature; and (3) develop recommendations for the acupuncture literature based on the REAL© results for the overall literature pool of reviews for each of the TSR components. The overall magnitude of effect of the results was not assessed nor calculated for the purposes of this review. SMEs performed the GRADE independently before discussing their answers together and coming to consensus.

Because some reviews included the same studies, the studies were de-duplicated across all reviews within each TSR component in order to determine the ‘true’ total number of studies and participants included. It is important to note, however, that some reviews differed in the way they calculated their total sample, and as such, the total number of participants we report may be slightly inaccurate; when there was a discrepancy between reviews in how they reported their total number of participants, the higher number of participants was taken. Further, while we present the total, de-duplicated, number of studies and participants in the GRADE and results, for purposes of this overall analysis, all studies were still included as reported in each review to describe each included review accurately.

## Results

**Figure 2:**
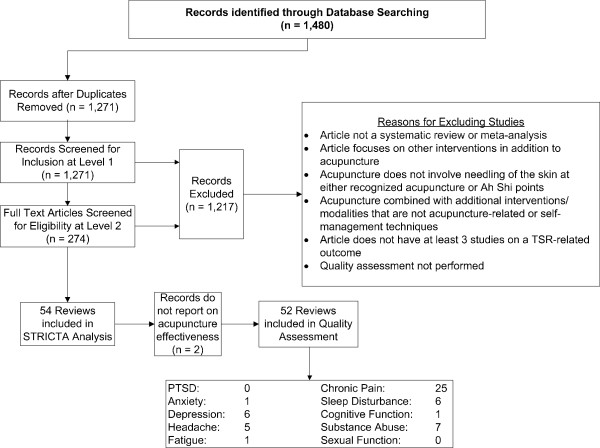
Flow chart of included studies.

Our search yielded a total of 1,480 citations from database inception to September 2011. Of the total 54 reviews included, 52 fit the inclusion criteria and were assessed using the SIGN 50 quality scoring tool for systematic reviews (see Figure 2 for the flow of included studies). Two studies [60,61] were excluded because they did not assess the effectiveness of acupuncture but instead focused on assessing STRICTA adherence and the methodology of included studies, and, as such, were included only in the STRICTA analysis.

Table 2 describes the characteristics of the individual reviews included as well as the overall SIGN 50 score, grouped by each TSR component; because this review focuses on the effectiveness of acupuncture, we have ranked reviews within each TSR component first by quality, and then by interpretation of results, so that an article’s results can be easily viewed within the context of its quality (see Additional file 1: Appendix 1 for SIGN 50 criteria and scores for each review). Table 3 describes the GRADE results of the overall literature pool and Table 4 describes the STRICTA assessment, both grouped by the individual TSR component.

**Table 2:** Characteristics and SIGN 50 score of included reviews grouped by TSR component

**Citation**	**Population**	**Type of acupuncture**	**Control**	**Outcomes relevant to TSR components**	**Number of relevant studies (total) included**	**Interpretation of results**	**How well was the study done to minimize bias?**
*Headache (n = 5)*							
Sun et al. [62]	3,916 subjects with chronic headache (migraine, tension-type or both)	electro	sham, pharmacologic, physiological, CAM	headache frequency/intensity, response rate	31(31) RCTs	Positive	++
Linde et al. [63]	1,151 subjects with migraine and tension-type headaches	traditional Chinese	no treatment, sham, pharmacologic, physiotherapy	headache diary, number of migraine days, migraine hours, frequency/duration of headache attack, headache severity, amount and type of rescue medication, nausea and vomiting frequency, pain intensity, number of pain days	22(22) RCTs	Positive	++
Linde et al. [64]	2,317 subjects with episodic and/or chronic tension-type headache	traditional Chinese	no treatment, sham, pharmacologic, physiological	analgesic usage, CGI, headache frequency/intensity/duration/location, global patient rating, PDI, VAS, CPG	11(11) RCTs	Positive	++
Melchart et al. [65]	(ND) subjects with recurrent headaches (tension-type, migraine, various)	manual, ear	sham, pharmacologic, physiological, no treatment, behavioral/psychosocial	headache attack frequency, global assessment of headache, number of days with headache, pain intensity, VAS	20(22) RCTs^a^	Positive, Poor methods	++
Granato et al. [66]	2,317 subjects with episodic or chronic tension-type headaches	manual	no treatment, sham, physiotherapy, CAM	frequency of analgesic use, number of headache days, VAS	11(11) RCTs	Positive	+
*Chronic pain (n = 25)*							
White et al. [67]	2,362 subjects with knee pain	manual, electro	sham, behavioral/psychosocial, no treatment, pharmacologic	WOMAC, pain scales	13(13) RCTs	Positive	++
Kwon et al. [68]	1,891 subjects with peripheral joint osteoarthritis	manual, electro	no treatment, sham, behavioral/psychosocial, physiotherapy, pharmacologic	VAS, WOMAC, MPQ, NRS, TUGT, HSS knee function scale, walking/climbing stairs time, starting pain, night pain, walking pain, pain descending stairs, pain threshold, Lysholm Score, PGA, present pain intensity, stiffness, active knee flexion, passive range of movement, total pain, effective rate, recurrence rate, Lequesne indices	18(18) RCTs	Positive	++
Manheimer et al. [69]	176 subjects with low back pain	Western	sham, physiotherapy, no treatment, acupressure, pharmacologic	VAS, drug use, fit for work score, global score, physician assessment of functionality	4(33) RCTs^b^	Positive	++
Fu et al. [70]	7,173 subjects with neck pain	manual, electro	sham, no treatment, physiotherapy, CAM	MPQ, NDI, PDI, ROM, self-reported pain, VAS	14(14) RCTs	Positive	++
Ezzo et al. [71]	393 subjects with knee osteoarthritis	electro, manual	sham, no treatment, physiotherapy	pain, patient global assessment, physical function	7(7) RCTs and quasi-RCTs	Positive	++
Wang et al. [72]	536 subjects with rheumatoid arthritis	manual, electro	sham, pharmacologic	CRP, DAS28, duration of morning stiffness, ESR, GHQ, HAQ, SJC, VAS	8(8) RCTs	Inconclusive/Mixed	++
Jung et al. [73]	134 subjects with temporomandibular joint disorders	manual	sham	MO, NRS, muscle tenderness, VAS	6(7) RCTs^a^	Inconclusive/Mixed	++
Manheimer et al. [74]	3,498 subjects with osteoarthritis of the knee and/or hip	manual	sham, no treatment, other	WOMAC	16(16) RCTs	Inconclusive/Mixed	++
La Touche et al. [75]	401 subjects with temporomandibular disorders	manual, electro	no treatment, sham	ADL, Anamnestic Index, Anamnestic Questionnaire, articular sounds/stereostethoscope, CDS, distribution of pain, index for occlusal state, incisal and occlusal tooth wear, maximum interincisal opening, NRS, pain frequency, PPT, subjective dysfunction score, VAS	8(8) RCTs	Positive, Poor methods	++
Cho et al. [76]	808 subjects with temporomandibular disorders	manual, electro	sham, no treatment, pharmacologic, physiotherapy	clinical dysfunction scores, NAS, ROM, sounds/locking/deviation in opening of mouth, tenderness, presence/absence of a headache, overall improvement, VAS	14(14) RCTs	Inconclusive/Mixed, Poor methods	++
Lee et al. [77]	606 subjects with rheumatoid arthritis	manual	sham, pharmacologic	ACR20, CRP, DAS, ESR, GHQ, HAQ, VAS, total effective rate, pain reduction, swelling index, number of swollen joints	8(8) RCTs	Inconclusive/Mixed, Poor methods	++
Sim et al. [78]	442 subjects with carpal tunnel syndrome	manual, laser	sham, pharmacologic, CAM	CMAP, D4MNSCV, D4UNSCV, DML, DSL, GSS, MNCV, NCS, night pain, parethesia, responder rate, SNAP, SSS, W-P SNCV	6(6) RCTs	Inconclusive/Mixed, Poor methods	++
Liu et al. [79]	506 subjects with trigeminal neuralgia	manual, electro	pharmacologic	cure rates	12(12) RCTs	Inconclusive/Mixed, Poor methods	++
Langhorst et al. [80]	385 subjects with fibromyalgia syndrome	manual, electro	sham	FIQ, MPQ, VAS	7(7) RCTs	Inconclusive/Mixed, Poor methods	++
Zhu et al. [81]	67 subjects with endometriosis	ear	CAM	dysmenorrhea score, therapeutic effect	1(1) RCT	Negative, Poor methods	++
van Tulder et al. [82]	542 subjects with chronic lower back pain	manual, electro	sham, no treatment, pharmacologic	ADL, global improvement, Lasoque test, pain score, Schober Test, VAS	11(11) RCTs	Negative, Poor methods	++
ter Riet et al. [83]	(ND) subjects with chronic pain	manual	sham, pharmacologic, acupuncture	ND	51(51) CCTs	Negative, Poor methods	++
Mayhew et al. [84]	166 subjects with fibromyalgia	traditional Chinese, electro	sham, no treatment, other	analgesics usage, dolorimetry of tender and control points, FIQ, morning stiffness, MPI, regional pain score, VAS	5(5) RCTs	Negative, Poor methods	++
Porter et al. [85]	486 subjects with fibromyalgia	electro, manual	sham, no treatment, pharmacologic	physical, psychological and quality of life outcomes	7(7) RCTs, 2(2) CCTs	Positive	+
Ernst et al. [86]	(ND) subjects with chronic pain	ND	manual, electro, ear	ND	30(30) SRs	Positive	+
White et al. [87]	2,362 subjects with osteoarthritis of the knee	electro, manual	sham, no treatment, physiologic, pharmacologic	NRS, PPI, VAS, WOMAC	13(13) RCTs	Positive	+
La Touche et al. [88]	83 subjects with temporomandibular disorders	manual	sham	electronic axiography, incisor opening and lateral movement, manual palpitation, NRS, pain distribution, pressure algometer, temporomandibular joint sounds/stereo-stethoscope, VAS	4(4)RCTs	Inconclusive/Mixed	+
Ernst [89]	437 subjects with osteoarthritis	manual, electro	sham, no treatment, pharmacologic, physiotherapy	analgesic usage, functioning, knee pain threshold, self-reported pain ratings, subjective improvement and ROM, tenderness, VAS	13(13) RCTs	Inconclusive/Mixed	+
Manheimer et al. [90]	1,154 subjects with osteoarthritis	manual, electro	sham, no treatment	WOMAC	11(11) RCTs	Inconclusive/Mixed	+
Casimiro et al. [91]	84 subjects with rheumatoid arthritis	manual, electro	sham, pharmacologic	analgesic usage, CRP, ESR, pain reduction, number of swollen/tender joints, VAS	2(2) RCTs	Inconclusive/Mixed, Poor methods	+
*Substance abuse (n = 7)*							
Cho et al. [92]	1,062 subjects with alcohol dependence	ear, ear electro	no treatment, sham, pharmacologic, non-pharmacologic	abstinent rate, alcohol usage, AWSS, breath analyzer, CIWA, completion rates, relapse rates	10(11) RCTs^a^	Inconclusive/Mixed, moor Methods	++
Gates et al. [93]	1,433 subjects with cocaine or crack dependence	ear	sham, no treatment	ASI, cocaine use, HCCS, urine toxicology	7(7) RCTs	Negative, Poor methods	++
White et al. [94]	3,486 subjects with tobacco addiction	manual, electro	sham, no treatment, behavioral/psychosocial, pharmacologic	complete smoking cessation	14(14) RCTs	Negative, Poor methods	++
D'Alberto et al. [95]	1,356 subjects with cocaine abuse or dependence	ear	sham, CAM	CCQ, urine toxicology	6(6) RCTs	Inconclusive/Mixed	+
White et al. [96]	1,433 subjects with smoking addiction	ear, electro	behavioral/psychosocial, pharmacologic, no treatment, acupuncture, CAM	quit rates	13(13) RCTs	Inconclusive/Mixed	+
ter Riet et al. [97]	(ND) subjects addicted to cigarette smoking, heroin, or alcohol	manual, electro	ND	acupuncture effectiveness	22(22) CCTs	Negative	+
Mills et al. [98]	1,747 subjects with cocaine addiction	ear	CAM, pharmacologic, behavioral/psychosocial	ASI, frequency/amount of cocaine use, HCCS, HDIRS, self-reported effectiveness, treatment effects, urine assays	9(9) RCTs	Negative	+
*Sleep disturbance (n = 6)*							
Cao et al. [99]	3,811 subjects with insomnia	rolling, scalp, ear, abdominal	no treatment, CAM, sham, pharmacologic	duration and quality of sleep, MQ, PFS, PSQI, SDRS, sleep quality, SSDS, SRSS, VAS	46(46) RCTs	Positive	++
Chen et al. [100]	673 subjects with insomnia	ear	no treatment, sham, pharmacologic	actigraphic monitoring, ISI, MQ, PSQI, sleeping hours	6(6) RCTs	Inconclusive/Mixed	++
Yeung et al. [101]	1,956 subjects with insomnia	manual	sham, no treatment, pharmacologic	NRS, MQ, ISI, AIS, PSQI, SDRS, SSDS, PSG, wrist actigraph monitoring, overnight polysomnogram, VAS	20(20) RCTs	Inconclusive/Mixed	++
Huang et al. [102]	1,355 subjects with insomnia	manual, ear, intradermal, rolling	sham, pharmacologic, education	AIS, ISI, PSQI, PFS, sleep diary, sleep time, wrist actigraph monitoring	2(3) RCTs, 8(9) CCTs, 18(18) case series^a^	Positive, Poor methods	++
Cheuk et al. [103]	300 subjects with insomnia	electro, traditional Chinese, contemporary	no treatment, sham	self-rated insomnia scale, sleep disturbance on numerical scale, ISI, AIS, MQ, actigraphy monitoring	4(7) RCTs^a^	Positive, Poor methods	++
Lee et al. [104]	842 subjects with insomnia	ear (needle, SV seeds taping, magnetic pearls)	sham, no treatment, pharmacologic	NST, Pittsburgh Sleep Diary, sleep efficiency, sleep quality, Karolinska Sleep Diary, 4-point Sleep Score, self-satisfaction scale	10(10) RCTs	Inconclusive/Mixed, Poor methods	+
*Depression (n = 6)*							
Zhang et al. [105]	1,680 subjects with MDD and PSD	manual, electro, ear	sham, no treatment, pharmacologic	HAM-D	35(35) RCTs	Positive	++
Fan et al. [106]	2,757 subjects with depression or depressive disorders	manual	sham, pharmacologic	CGI, DSI, efficacy rate, HAM-D, SCL-90, SDS	19(20) RCTs^a^	Positive	++
Mukaino et al. [107]	509 subjects with depression	manual, electro	no treatment, sham, pharmacologic	BRMS, CGI, HRSD	7(7) RCTs	Inconclusive/Mixed	++
Wang et al. [108]	447 subjects with major depression or depressive neurosis	manual, electro	sham	HAM-D	7(8) RCTs^a^	Inconclusive/Mixed, Poor methods	++
Smith et al. [109]	2,782 subjects with depression	abdominal, manual	sham, no treatment, pharmacologic	assessment of improvement, BDI, CGI, cure rates, HAM-D, HRSD, medication usage, Melancholia Scale, remission rates	29(30) RCTs^a^	Inconclusive/Mixed, Poor methods	++
Leo et al. [110]	666 subjects with depression	verum, manual, ear, traditional Chinese, electro	no treatment, sham, CAM	BDI, CES-D, CGI, HAM-D	9(9) RCTs	Inconclusive/Mixed	+
*Anxiety (n = 1)*							
Pilkington et al. [111]	1,201 subjects with anxiety or an anxiety disorder	traditional Chinese, Western medical, ear, electro	sham, behavioral/psychosocial, pharmacologic	anesthesia dose, CGI, cure rates, HAM-A, MMPI, MYPAS, patient/observer assessment of anxiety, STAI, VAS, X-1	8(10) RCTs, 2(2) CCTs^a^	Positive	++
*Cognitive function (n = 1)*							
Zhao et al. [112]	960 subjects with vascular dementia	manual, electro, targets, ear	sham, pharmacologic	ADL, FAQ, HDS, MMSE-R, overall function	9(10) RCT, 1(1) quasi- RCT^a^	Inconclusive/Mixed, Poor methods	++
*Fatigue (n = 1)*							
Wang et al. [113]	1,826 participants with chronic fatigue syndrome	manual, electro	pharmacologic, CAM	improvement in chronic fatigue symptoms, FAI, SCL-90, CFIDS Disability Scale	22(27) Clinical Trials, 9(13) RCTs^a^	Positive, Poor methods	***_***

^a^Some studies included in the reviews were excluded because they did not utilize manual acupuncture at recognized acupuncture points (that is, laser acupuncture, acupressure, needling of trigger points); ^b^number of chronic pain studies may actually be higher; review only reported number of chronic pain studies not combined in meta-analysis instead of total number of chronic pain studies. Acu, acupuncture; AWSS, Alcohol-Withdrawal Syndrome Scale; ACR20, American College of Rheumatology Criteria; ADL, Activities of Daily Living; ASI, Addiction Severity Index; AIS, Athens Insomnia Index; BDI, Beck Depression Inventory; BRMS, Beck Melancholia Scale; CCT; controlled clinical trial; CCQ, Cocaine Craving Questionnaire; CCS, clinical case series; CDS, Clinical Dysfunction Score; CIWA, Clinical Institute Withdrawal Assessment; CES-D, Center for Epidemiological Study of Depression; CFIDS Disability Scale, Chronic Fatigue/Immune Dysfunction Syndrome Disability Scale; CPG, von Korff Chronic Pain Grading Scale; CGI, clinical global impression; CMAP, compound muscle action potential; CRP, C-reactive protein; D4MNSCV, 4th digit median nerve sensory nerve conduction velocity; D4UNSCV, 4th digit ulnar nerve sensory nerve conduction velocity; DAS, Disease Assessment Scale; DAS28, Disease Activity Scale in 28 Joints; DML, distal motor latency; DSI, Depression Status Inventory; DSL, Distal Sensory Latency; ESR, erythrocyte sedimentation rate; FAI, Fatigue Assessment Instrument; FAQ, Functional Activities Survey; FIQ, Fibromyalgia Impact Questionnaire; GHQ, General Health Questionnaire; GSS, Global Symptom Score; HDIRS, Halikas Drug Impairment Rating Scale; HAM-A, Hamilton Anxiety Scale; HAM-D, Hamilton Depression Rating Scale; HAQ, Health Assessment Questionnaire; HDS, Hasegawa Dementia Scale; HRSD, Hamilton Rating Scale for Depression; HSS, Hospital for Special Surgery; ISI, Index of Severity of Insomnia; JOA, Japanese Orthopedic Association Measure; MDD, Major Depressive Disorder; MMPI. Minnesota Multiphasic Personality Inventory; MMSE, Mini Mental State Examination; MNCV, motor nerve conduction velocity; MPI, Multidisciplinary Pain Inventory; MPQ, McGill Pain Questionnaire; MQ, Morning Questionnaire; MYPAS, Modified Yale Preoperative Anxiety Scale; NCS, Nerve Conduction Studies; ND, not described; NDI, Neck Disability Index; NAS, Numerical Analog Scale; NRS, Numeric Rating Scale; NST, nocturnal sleep time; ODI, Oswestry Disability Index; PDI, Pain Disability Index; PGA, Patient Global Assessment; PFS, Piper Fatigue Scale; PPI, Present Pain Index; PPT, pressure pain threshold; PSD, post-stroke depression; PSG, polysomnography; PSQI, Pittsburgh Sleep Quality Index; RCT, randomized controlled trial; ROM, range of motion; RDS, Roland Disability Score; SCL-90, Symptom Checklist-90; SDRS, Sleep Dysfunction Rating Scale; SDS, Self-rating Depression Scale; SJC, swollen joint count; SNAP, sensory nerve action potential; SRSS, Self-Rating Sleep Scale; SSDS, Self-Rating Sleep Dysfunction Scale; SSS, Symptom Severity Score; STAI, State-Trait Anxiety Inventory; TUGT, Timed Up and Go Test; VAS, Visual Analogue Scale; WOMAC, Western Ontario MacMaster Osteoarthritis Index; W-P SNCV, Wrist Palm Sensory Nerve Conduction Velocity.

Timed Up and Go Test; VAS, Visual Analogue Scale; WOMAC, Western Ontario MacMaster Osteoarthritis Index; W-P SNCV, Wrist Palm Sensory Nerve Conduction Velocity.

**Table 3:** **TSR GRADE table**: **quality in the overall literature pool by TSR components for acupuncture**

**TSR component**	**Total number of reviews**	**Number of studies** (**total number of participants**)	**Confidence in the results**	**Safety GRADE**	**GRADE recommendation**
Chronic pain	25	163 (12,675)^a^	Acupuncture promising, but no conclusions yet	+1^c^	weak recommendation in favor
Sleep disturbance	6	83 (9,623)^b^	Acupuncture promising, but no conclusions yet	+1^c^	weak recommendation in favor
Depression	6	73 (9,986)	Acupuncture promising, but no conclusions yet	0^c^	weak recommendation in favor
Headache	5	53 (8,274)^b^	Acupuncture shown to be effective	0^c^	weak recommendation in favor
Anxiety	1	10 (1,201)	Acupuncture promising, but no conclusions yet	0	weak recommendation in favor
Substance abuse	7	48 (7,433)^b^	Acupuncture shown to be not effective	+1^c^	weak recommendation against
Cognitive function	1	10 (960)	Unable to interpret/contradictory results	N/A^c^	no recommendation
Fatigue	1	31 (1,826)	Unable to interpret/contradictory results	+1	no recommendation

NOTE: Studies included within each review were de-duplicated. Because there was some discrepancy between reviews in how they reported their total number of participants, it is possible that the number of participants is slightly inaccurate; for purposes of this review, all studies were included as reported in each review; ^a^number of participants not described in two studies; ^b^number of participants not described in one study; ^c^studies that did not report on adverse events were not included in the safety grade.

There are three major domains that comprise the core of the modified GRADE methodology: 1) Confidence in the results was categorized into the following groups using pre-defined criteria: (1) Acupuncture shown to be effective: the majority of the results are of high quality and all show positive results; or there is a most recent largest review showing positive results of highest quality; (2) Acupuncture promising, but no conclusions yet: mix of positive and inconclusive results, but no negative results found among the reviews; the majority of the reviews are of high quality; (3) Unable to interpret/contradictory results: low quality review or the majority of the studies have mixed/inconclusive results; or (4) Acupuncture shown to be not effective: the majority of the reviews report negative results. 2) Safety grade is dependent on the frequency and severity of adverse events and interactions. Safety is categorized into one of the following grades: +2: appears safe with infrequent adverse events and interactions; +1: appears relatively safe but with frequent but not serious adverse events and interactions; 0: safety not well understood or conflicting; -1: appears to have safety concerns that include infrequent but serious adverse events and/or interactions; or −2: has serious safety concerns that include frequent and serious adverse events and/or interactions. 3) Strength of the recommendation can be determined using the following categories and criteria: Strong recommendation in favor of or against: very certain that benefits do, or do not, outweigh risks and burdens; No recommendation: no recommendations can be made; or Weak recommendation in favor of or against: benefits and risks and burdens are finely balanced, or appreciable uncertainty exists about the magnitude of benefits and risks.

**Table 4:** STRICTA analysis

	**Headache** (**6**)	**Chronic pain** (**26**)	**Substance abuse** (**7**)	**Sleep disturbance** (**6**)	**Depression** (**6**)	**Anxiety** (**1**)	**Cognitive function** (**1**)	**Fatigue** (**1**)	**TOTAL** (**54**)^**a**^
Acupuncture rationale	6/6	24/26	7/7	6/6	6/6	1/1	1/1	1/1	96%
Details of needling	4/6	18/26	5/7	4/6	3/6	1/1	0/1	1/1	67%
*Treatment regimen*									
Number of treatment sessions	4/6	16/26	4/7	5/6	4/6	1/1	0/1	1/1	65%
Frequency of treatments	2/6	9/26	5/7	4/6	1/6	0/1	1/1	0/1	41%
*Other components of treatment*									
Other components	6/6	23/26	6/7	6/6	6/6	1/1	1/1	1/1	93%
Setting and context	3/6	0/26	0/7	0/6	0/6	0/1	0/1	0/1	6%
Practitioner background	4/6	1/26	3/7	0/6	1/6	0/1	0/1	0/1	2%
Control	6/6	25/26	6/7	6/6	6/6	1/1	1/1	1/1	96%
Total	73%	56%	64%	65%	56%	63%	50%	63%	

^a^Two References [60,61] did not assess effectiveness of acupuncture and were excluded from outcomes assessment but included in STRICTA analysis.

There are three major domains that comprise the core of the modified GRADE methodology: 1) Confidence in the results was categorized into the following groups using pre-defined criteria: (1) Acupuncture shown to be effective:the majority of the results are of high quality and all show positive results; or there is a most recent largest review showing positive results of highest quality; (2) Acupuncture promising, but no conclusions yet: mix of positive and inconclusive results, but no negative results found among the reviews; the majority of the reviews are of high quality; (3) Unable to interpret/contradictory results: low quality review or the majority of the studies have mixed/inconclusive results; or (4) Acupuncture shown to be not effective: the majority of the reviews report negative results. 2) Safety grade is dependent on the frequency and severity of adverse events and interactions. Safety is categorized into one of the following grades: +2: appears safe with infrequent adverse events and interactions; +1: appears relatively safe but with frequent but not serious adverse events and interactions; 0: safety not well understood or conflicting; -1: appears to have safety concerns that include infrequent but serious adverse events and/or interactions; or −2: has serious safety concerns that include frequent and serious adverse events and/or interactions. 3) Strength of the recommendation can be determined using the following categories and criteria: Strong recommendation in favor of or against: very certain that benefits do, or do not, outweigh risks and burdens; No recommendation: no recommendations can be made; or Weak recommendation in favor of or against: benefits and risks and burdens are finely balanced, or appreciable uncertainty exists about the magnitude of benefits and risks.

### Effectiveness of acupuncture for TSR components

#### Headache

Five reviews [62-66] of high quality (++ or +), involving 53 total RCTs and 8,274 participants with headaches of varying etiologies, reported consistently favorable effects. Although safety was not clearly assessed in the included reviews, the literature suggests that acupuncture is effective for headache. Because safety is not well documented in these reviews, we believe there can only be a weak recommendation in favor of acupuncture in this area and that more high powered studies that report on safety of acupuncture are needed for this condition.

#### Chronic pain

Twenty-five reviews [67-91], involving 162 RCT and controlled clinical trials (CCT) and one review of reviews with 12,675 patients total, assessed acupuncture efficacy in treating a range of chronic pain conditions (as defined in Table 1) including low back pain, neck pain, osteoarthritis, knee pain, temporomandibular disorders, carpel tunnel syndrome, rheumatoid arthritis, and trigeminal neuralgia. The majority of the results reported were either inconclusive/mixed [72-74,76-80,88-91] or positive [67-71,75,85-87] with only four reviews [81-84] reporting negative acupuncture effectiveness. We characterize acupuncture as ‘promising for pain but with no conclusions able to be drawn’ at this point because, although all the reviews were high quality, most of the reviews reported mixed results. Because acupuncture appears to be relatively safe for this condition, a weak recommendation can be made in favor of acupuncture for general chronic pain treatment. More high powered studies are needed to increase our confidence to a strong recommendation.

#### Substance abuse

Seven high quality reviews [92-98] involving 48 RCTs and CCTs and 7,433 participants, studied populations with different types of substance abuse problems, including alcohol, cocaine, crack, and nicotine dependencies and other addictions. Overall, acupuncture was reported as not effective in treating these conditions. The majority of reviews had either negative or inconclusive/mixed results. Although acupuncture appears to be relatively safe, with frequent but non-serious adverse events, acupuncture is not recommended for the treatment of substance abuse problems based on the amount of negative and inconsistent results found in this literature pool. A weak recommendation against acupuncture for substance abuse is suggested based on the current literature available.

#### Sleep disturbance

All reviews [99-104], including 9,623 patients across 83 RCT and CCTs, with insomnia, were of high quality. Half of the reviews reported positive results [99,102,103] for acupuncture while the remainder reported inconclusive/mixed results [100,101,104] suggesting that acupuncture may be a promising treatment option. Based on this literature pool, acupuncture appears to be relatively safe, and, as such, a weak recommendation is made for the treatment of sleep disturbances, although no firm conclusions can be drawn yet.

#### Depression

Six high quality reviews [105-110] including 73 RCTs with 7,986 participants assessed the efficacy of acupuncture in treating patients with depression or depressive disorders. Four reviews reported inconclusive/mixed results and two reviews [105,106] documented positive acupuncture effects. These two reports included the two most recent reviews on acupuncture for depression involving 35 and 19 studies, respectively. Although safety was not clearly assessed in these reviews, acupuncture seems to be a promising treatment, but no definitive conclusions can be drawn at this point. A weak recommendation can be made in favor of this intervention for depression; however, more studies assessing safety issues are needed.

#### Anxiety

One review of high quality [111] reported positive results in a population of 1,201 patients across eight RCT and two CCTs with anxiety or anxiety disorders. Safety was not clearly assessed in this review across the studies. Acupuncture is promising for this condition due to the consistently positive results but because safety is not well understood at this point, only a weak recommendation can be made in favor. More high powered studies are needed in this area.

#### Cognitive function

Reviews on cognitive processes involving memory, attention, concentration and problem-solving were searched. Only one high quality review [112] involving 10 RCTs and 960 participants was found, reporting inconclusive/mixed acupuncture results for treating subjects with vascular dementia. Given that adverse events were not reported and results were unable to be interpreted, no recommendation can be made as to whether or not acupuncture is a viable treatment option for cognitive functioning.

#### Fatigue

Although one review [113] including 31 CCT and RCT studies involving a population of 1,826 participants with chronic fatigue syndrome showed positive acupuncture results and frequent, but non-serious adverse events, the quality of this review was rated low and, therefore, no recommendation can be made for acupuncture at this time.

There were no reviews on acupuncture and PTSD or sexual function that met the pre-defined inclusion criteria.

### STRICTA and safety assessment

Overall, the majority of reviews addressed acupuncture rationale (96%), control or comparator interventions (96%) and details of other interventions administered to the acupuncture group (93%). A large number of reviews also reported on details of needling (67%) and aspects of the treatment regimen including number of treatment sessions (65%) and frequency of treatments (41%). Settings and context of treatment (6%) as well as practitioner background (2%) were the least reported STRICTA components. Further, the reviews included within each TSR component covered a similar percentage of STRICTA criteria (ranging from 50% to 65%) with the headache reviews covering the majority of STRICTA criteria (73%; see Table 4).

Expected, likely risks reported from acupuncture include localized discomfort and infection, slight bleeding and bruising at needle insertion sites [114-116]. Serious adverse events (that is, injury to brain/spinal cord, pneumothorax, cardiac tamponade, systemic infection, syncope) have been reported but are extremely rare [114]. Of the 52 reviews included in our analyses, 29 [63,70,71,73,74,76-79,84,85,90,92,93,95,98-105,107-111,113] reported on adverse events with six [53,62,70,73,84,93,95] reviews reporting no adverse events. Twelve reviews [74,76,85,90,92,98,101-105] reported expected, likely adverse events, and four reviews [63,70,78,111] reported that adverse events occurred but did not describe them. A number of reviews also reported more uncommon events including dizziness/fainting [77,79,104,105,113], drowsiness/fatigue [92,99,108], gastrointestinal problems [77,104,105], sleep disturbance [102,109], dry mouth [104,109], and headache [99,104,109]. Other adverse events such as herpes zoster [77], ‘blunt sight’ [104], hangover [104], general weakness [104], failure of coordination [104], palpitations [109] and ataxia [79] were less frequently reported with each of these events only being reported once in a single review.

## Discussion

Although more quality research and safety information are needed to determine whether acupuncture is useful in treating TSR components (.that is, PTSD, sexual function, cognitive function, fatigue), the results of our review suggest that acupuncture may be a beneficial or promising treatment for some components (that is., headache, anxiety, sleep disturbances, depression, chronic pain) and not beneficial for others (that is, substance abuse).

When reviewing the data in total, a number of factors influenced our conclusions and subsequent recommendations. The years in which the individual reviews were conducted had an impact on quality as the majority of the studies were conducted in the early to mid-2000s and at that time the number of quality clinical trials was low. There has been a sizable increase in the number of acupuncture studies of higher quality within the last decade. Thus, more recent reviews are more likely to capture accurately the effectiveness of acupuncture for TSR components [117].

In addition, there has been improvement in the reporting of acupuncture trials since the 2001 STRICTA publication. Yet, based on the 2010 revision of the STRICTA guidelines, significant inconsistency remains in the quality of reporting around the acupuncture intervention studies. Moreover, journals do not regularly require standardized reporting guidelines and those that do are not rigorous in ensuring that the guidelines are followed. [58] Although a number of the quality criteria were addressed in these reviews, STRICTA components should be more consistently applied in future studies and by journals in order to increase our confidence that acupuncture studies are being performed and reported properly.

We also examined the data for any reports about the setting, context and expectations of treatment and found that information on these aspects of the studies was addressed in only 6% of the reviews. Context, ritual and expectation have a significant impact on clinical outcomes in acupuncture trials [118-121], and these are likely to be important aspects to consider particularly when conducting an acupuncture trial in a physically and psychologically traumatized population. Creating an empathic, healing relationship [122] and helping patients find ‘embodied’ states of safety prior to placement of acupuncture needles may enhance its effectiveness, mitigate anxiety and pain associated with needle placement, and deepen the critical experience of safety for trauma survivors. We recommend that future research collect and report on these aspects. This will improve our understanding about what and how much of the effects of acupuncture are attributable to ritual, beliefs and other placebo components versus the specific effects of acupuncture [123].

Overall, there were few highly powered studies included in the reviews and most reviews did not report on adverse events. While acupuncture has been shown to be generally safe [115], safety was not described in the majority of reviews, making it difficult to provide a strong GRADE recommendation when this information was lacking. Thus, the majority of conditions were given a ‘weak recommendation in favor’ of acupuncture. Future research should address safety reporting, and detail this well enough for the reader to understand fully all acupuncture-related adverse events.

For a number of the TSR components, particularly chronic pain, there was a notable diversity of conditions listed in the individual reviews. It was difficult for us to tease out chronic pain conditions in these reviews that fit our pre-determined definition for chronic pain according to the American Chronic Pain Association’s criteria. Despite this existing diversity, the tools utilized for the individual quality assessment of the reviews allowed for informed conclusions to be made. Formulating more narrowly focused research questions in particular conditions, however, may help researchers better understand the treatment effects of acupuncture for given populations within specific conditions and is a topic for future research.

This review had a few limitations. First, the number of participants for each TSR component may not be completely accurate because some reviews included the same studies. While we were able to identify which studies were duplicated across reviews, many reviews differed in the way in which they reported their total participants; this discrepancy prevented us from accurately determining the number of total participants. Second, we were unable to capture the magnitude of the effect because this information was not always reported in the included reviews for our outcomes of interest. Third, only systematic reviews that assessed methodological quality were considered and we subsequently excluded any narrative or descriptive reviews. The fourth study limitation was our adherence to pre-defined inclusion criteria. Because we applied our criteria to both the reviews and their included studies, we did not always include the full set of studies from each review as some of the reviews (that is, those on laser acupuncture) did not meet our criteria. Finally, we reviewed acupuncture across each individual TSR component rather than across all components because there is no current literature that assesses the entire TSR spectrum. Since it appears that acupuncture affects more of the TSR components, future research should examine the impact of symptoms, quality of life and function in combinations of these conditions such as in CMI. To our knowledge only one study has specifically examined the effectiveness of acupuncture in CMI [124,125].

Despite this lack of research on the TSR spectrum and CMI in its entirety, research focusing on this data suggests that acupuncture may build resiliency to these symptoms in returning service members. Walker and colleagues from the Mental Health and Behavioral Sciences Service at the Veterans Affairs Hospital in Tampa, FL have noted that the common triad of post-concussive syndromes, chronic pain and PTSD symptoms is rather unique to the OEF/OIF population and have characterized its multi-symptom presentation as ‘Post-Deployment Multi-Symptom Disorder’ [126]. Acupuncture may be a common approach to this and CMI in other non-OEF/OIF veterans.

This multi-component and overlapping constellation of injuries in returning warfighters, also called War-related Trauma Spectrum Response (wrTSR), is very likely of different character and requires a different approach than civilian TSR [29]. One such difference is the capacity for what has recently been termed ‘moral injury’, defined as ‘perpetrating, failing to prevent or bearing witness to acts that transgress deeply held moral beliefs and expectations’ [127]. Because war and combat can create situations that affect this human dimension, future research should focus on identifying prevalence rates and possible co-morbid conditions to moral injury as this may be part of the TSR and other poly-trauma conditions such as Post-Deployment Multi-Symptom Disorder.

An adequate evaluation of acupuncture’s whole person impact on TSR requires a robust and comprehensive assessment that goes beyond compartmentalization of TSR into discrete disease states and acupuncture into uniformly prescribed treatment protocols. In our review, we detail a systematic and methodologically sound approach to assessing the level of evidence of acupuncture for the treatment of TSR that reflects its manifestation– as a set of complex and overlapping physical and psychological symptoms- and by doing so we provide a more complete picture of acupuncture’s effectiveness and the quality of acupuncture research, as it exists in the literature, than any one review on a single component could provide alone. Further, while the approach of this review allowed some recommendations to be made, these should be considered within the context of the individual reviews from which they were generated and not as practice guidelines. This review does, however, provide a basis for development of clinical guidelines and use by future expert panels and policy makers when making recommendations about appropriateness for use. Future research should begin to look across the multi-symptom clusters of TSR and CMI and explore acupuncture for its effectiveness and safety when treating whole person.

## Conclusions

This is the first review of reviews that explores acupuncture’s effectiveness in treating components of the TSR. Based on the results of our review, acupuncture has demonstrated benefit for the treatment of headaches; however, safety needs to be more fully documented in order to make any strong recommendations in support of its use in treating headaches. Though more research is needed to determine whether acupuncture is useful in treating anxiety, sleep disturbances, depression and chronic pain, it does seem to be a promising treatment option. Based on our results, acupuncture does not seem to be effective for treating substance abuse, and there needs to be more high quality data before we can determine whether acupuncture is an appropriate intervention for fatigue or cognitive difficulties.

## Abbreviations

CAM: complementary and alternative medicine; CCT: controlled clinical trial; CMI: complex multi-symptom illness; GRADE: Grading of Recommendation Assessment Development and Evaluation; OEF: Operation Enduring Freedom; OIF: Operation Iraqi Freedom; PICOS: population intervention, control, study design; PTSD: post-traumatic stress disorder; REAL: Rapid Evidence Assessment of the Literature; SIGN50: Scottish Intercollegiate Guidelines Network; SME: subject matter expert; STRICTA: Standards for Reporting Interventions in Clinical Trials of Acupuncture; TBI: traumatic brain injury; TSR: trauma symptom response.

## Competing interests

The authors declare that they have no competing interests.

## Authors’ contributions

CL and CC conceived and designed the study, gathered, analyzed and interpreted the data, and drafted the manuscript. DW, RW and WJ contributed to the conception of the study design, interpretation of data and manuscript preparation. AY, AD, JS and MS contributed to the acquisition and analysis of data as well as drafting of the manuscript. All authors have read and approved the final manuscript.

## Disclosures

The authors have not presented this data and information before in any journal or presentation and have no professional relationships with companies or manufacturers who will benefit from the results of this present study. This material is based upon work supported by the US Army Medical Research and Materiel Command under Award No. W81XWH-06-1-0279. Any opinions, findings and conclusions or recommendations expressed in this material are those of the author(s) and should not be construed as an official Department of the Army position, policy or decision unless so designated by other documentation.

## Supplementary Material

Additional file 1 **Appendix 1.** SIGN 50 Criteria and Scores. Click here for file
