# Supplementary material for: The effectiveness of acupuncture research across components of the trauma spectrum response (tsr): a systematic review of reviews
Source: Syst Rev. 2012 Oct 15;1:46. doi: 10.1186/2046-4053-1-46 (PMC3534620; doi:10.1186/2046-4053-1-46)
Supplement: Supplementary file 1 [file 2046-4053-1-46-S1.doc]

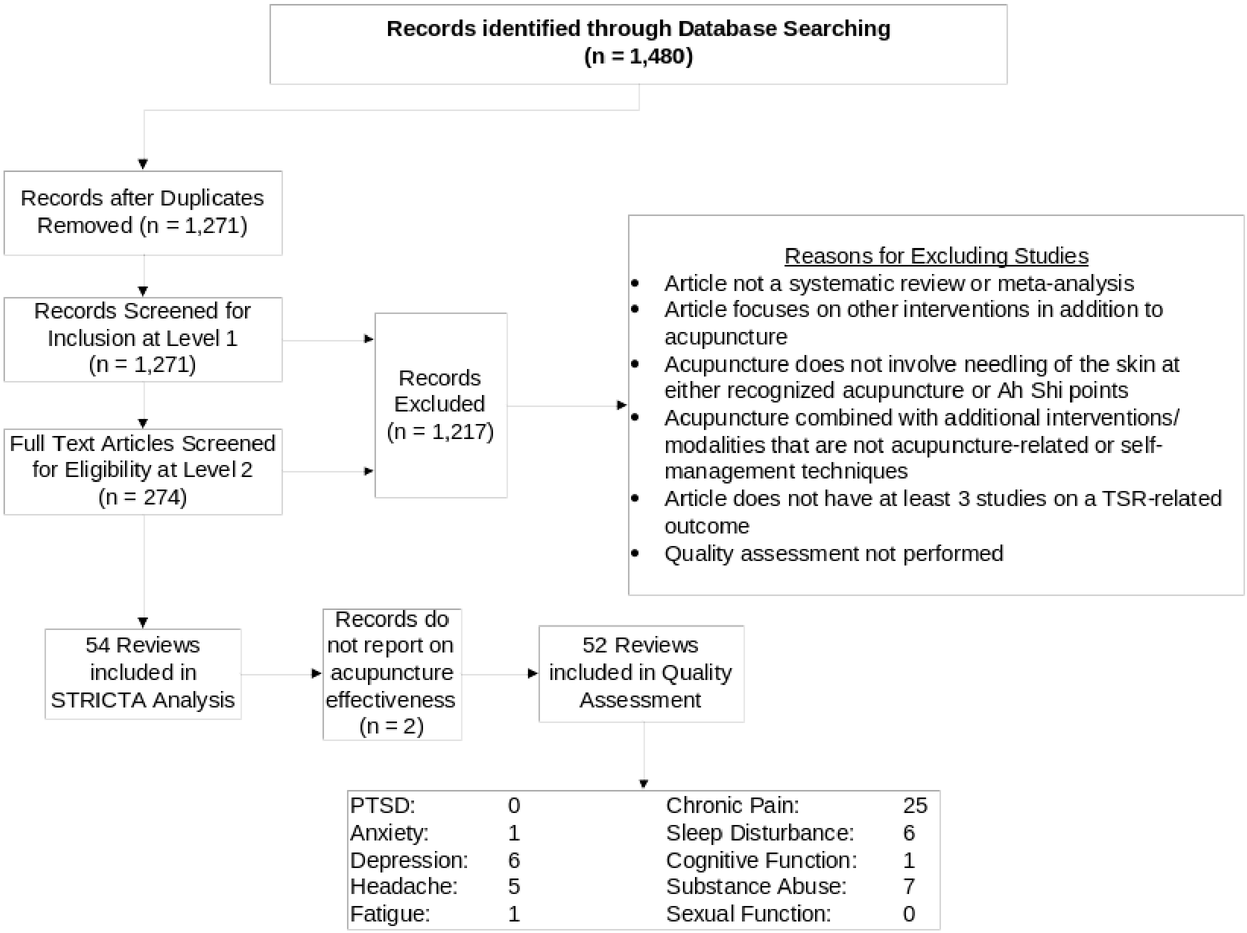
Records identified through Database Searching
(n = 1,480)
Records after Duplicates Removed (n = 1,271)
Records Screened for Inclusion at Level 1
(n = 1,271)
Full Text Articles Screened for Eligibility at Level 2
(n = 274)
Records Excluded
(n = 1,217)
Reasons for Excluding Studies
• Article not a systematic review or meta-analysis
• Article focuses on other interventions in addition to acupuncture
• Acupuncture does not involve needling of the skin at either recognized acupuncture or Ah Shi points
• Acupuncture combined with additional interventions/ modalities that are not acupuncture-related or self-management techniques
• Article does not have at least 3 studies on a TSR-related outcome
• Quality assessment not performed
54 Reviews included in STRICTA Analysis
Records do not report on acupuncture effectiveness
(n = 2)
52 Reviews included in Quality Assessment
PTSD: 0
Anxiety: 1
Depression: 6
Headache: 5
Fatigue: 1
Chronic Pain: 25
Sleep Disturbance: 6
Cognitive Function: 1
Substance Abuse: 7
Sexual Function: 0
